# Malnutrition and Gut Flora Dysbiosis: Specific Therapies for Emerging Comorbidities in Heart Failure

**DOI:** 10.1155/2015/382585

**Published:** 2015-09-30

**Authors:** Evasio Pasini, Roberto Aquilani, Giovanni Corsetti, Francesco S. Dioguardi

**Affiliations:** ^1^“S. Maugeri Foundation” IRCCS, Medical Centre, Lumezzane, 25065 Brescia, Italy; ^2^Department of Biology and Biotechnology “L. Spallanzani”, University of Pavia, 27100 Pavia, Italy; ^3^Division of Human Anatomy and Physiopathology, Department of Clinical and Experimental Sciences, University of Brescia, 25124 Brescia, Italy; ^4^Department of Internal Medicine, University of Milan, 20100 Milan, Italy

## Abstract

Chronic heart failure is a complicated multifactorial disease with wide-spread social-economic consequences. In spite of the recent development of new drugs and therapeutic strategies, CHF-related mortality and morbidity remain high. Recent evidence suggests that changes in organs such as skeletal muscle and gut flora may play an important and independent role in CHF prognosis. This paper illustrates these phenomena, proposing how to identify them and presenting current therapies which treat organs all too often underestimated but which have a fundamental role in worsening CHF.

## 1. Introduction

Chronic heart failure (CHF) is a very common and multifactor syndrome. In spite of currently used therapies, it still has high mortality and mobility [1]. Malnutrition, in particular with protein metabolism impairment and consequent cachexia, has often been found in CHF patients and correlates closely with mortality [2]. Furthermore, we recently demonstrated that CHF patients have important changes in both gut flora and intestinal permeability, which influence systemic inflammation and global metabolism by modifying nutrients absorption and production of molecules fundamental for cell life (authors' unpublished data). These conditions are often ignored or underestimated by most clinicians, although they have been shown to increase morbidity, hospital stay, and mortality risk independent of the primary cause of CHF.

This paper illustrates the clinical problems related to malnutrition (basically, protein malnutrition) and gut flora modification in CHF patients. We also look at some possibly easily repeatable methods to identify these pathological conditions and to try to care for them. In the wait for genetic therapies and stem cells for CHF [3], we believe that identifying and possibly curing protein malnutrition and composition alteration and/or gut microbiota activity could be an important step to improve CHF patient care. This would avoid additional and independent clinical damage and allow traditional therapies to work more efficiently.

## 2. Malnutrition

### 2.1. A Definition

Malnutrition is a general term that encompasses various forms of inadequate nutrition. It is multifactorial but is basically caused by the reduced balance between body nutrient intake and needs. Qualitative and quantitative malnutrition are often present in CHF and protein malnutrition, leading to the disarrangement of both visceral and muscular proteins. These conditions have been extensively studied in CHF [2, 4].

Protein malnutrition can be estimated in patients by measuring visceral protein (e.g., serum albumin concentrations) and muscular wasting (e.g., arm muscle area). Indeed, serum albumin level < 3.5 g/dL (without diseases interfering with albumin metabolism, such as liver or renal insufficiency) and muscular sarcopenia (<5th percentile of normal upper-arm muscle area) are important markers of poor protein nutritional status. This is because they can predict morbidity and mortality in CHF independent of the primary cause of the disease [5, 6]. Recently, other simple nutritional assessment tools, such as the Geriatric Nutritional Risk Index (GNRI), which considers serum albumin and body mass index (BMI), correlate with lower serum hemoglobin and systemic inflammation (higher C-reactive protein) and have been seen to be useful aids in CHF. This index predicts functional dependency and mortality in CHF patients even with a preserved ejection fraction [7].

### 2.2. The Clinical Problem

Data show that protein visceral malnutrition impairment in patients with CHF is not always as severe as muscle wasting. Very often, visceral protein synthesis is also conserved. Indeed, skeletal muscle wasting (measured by magnetic resonance spectroscopy) was found in 68% of CHF patients, while up to 24% of patients had serum albumin levels <3.5 g/dL [3]. This apparent discrepancy can be explained by considering the metabolic role of the amino acids (AA) of striate muscles. Indeed, we can say that skeletal muscle is a pivotal organ, which maintains body metabolic performance by the continuous exchange of fuel with the liver. Under malnutrition, starvation, and/or catabolic circulating stimuli, striate muscle protein is degraded and AA are released into the blood. The released AA are essential for maintaining global protein synthesis (including albumin) and glucose plasma levels, through hepatic gluconeogenesis and cell energetic metabolism. Indeed, AA contain carbon, oxygen, hydrogen, and nitrogen. They can therefore be metabolized into carbohydrates (such as glucose) or directly into lipids in accordance with the thermodynamic status of the cell. It must be pointed out that lipids cannot be transformed into carbohydrates by mammals. In addition, AA are fundamental intermediaries for the tricarboxylic acid cycle [8]. Clinically interesting, about 16% of patients with muscular wasting progress to cachexia. However, this phenomenon is related to a dramatic increase in mortality as 50% of cachectic patients die within 18 months [9, 10].

### 2.3. Why It Occurs

The genesis of visceral and muscular proteins breakdown in CHF has not yet been completely understood. The more accepted hypotheses indicate that the catabolic effects of inflammatory cytokines (e.g., tumor necrosis factors and interleukins) and neuroendocrine hormones (catecholamines, cortisol, and renin) are responsible for this phenomenon [11, 12].

Indeed, the increase in plasma catabolic molecules in patients with CHF has been well documented. Recently, impairment of anabolic hormones such as insulin with insulin resistance (IR) has also been found in 58% of CHF patients [13]. In muscles, the lack of anabolic stimulation due to IR and the increase of catabolic stimuli causes protein degradation and AA release. These AA are used in the liver to produce glucose by gluconeogenesis. In addition, IR inhibits mRNA synthesis of phosphoenol pyruvate carboxykinase (the key enzyme of the gluconeogenesis pathway) in the liver.

This contributes to reducing gluconeogenesis further via pyruvate originating from lactate. Thus, a vicious circle is generated, where malnutrition does not supply exogenous nutrients, the liver produces low quantities of carbohydrate from alternative metabolic pathways, and muscle AA are used to produce glucose essential to maintain the glucose-dependent metabolism of fundamental structures such as the brain and erythrocytes.

Moreover, under these conditions, muscle glycogen reserves are depleted and free fatty acids (FFA) become the principal fuel for the muscle. However, FFA use is a limiting factor for energy production [14]. In fact, accumulation of FFA in the myocytes reduces energy production during exercise with consequent skeletal muscle fatigue [15]. Notably, insulin also plays an important role in regulating adipose metabolism. Small increases in plasma insulin significantly reduce lipolysis in adipose tissue. This insulin increase reduces the availability of FFA for acetyl-CoA production, which is fundamental for maintaining cell energy production. Consequently, cell energy production via the anaerobic metabolic pathway is maintained by acetate which is derived predominantly from AA breakdown and oxidation. It is clear that the availability of AA is a key factor in maintaining mammal cell metabolism.

### 2.4. Possible New Therapeutic Approaches

In the light of these findings, we can say that the alteration of protein metabolism is clinically important. This could be due to different conditions related to age and/or inflammatory diseases, reduced qualitative/quantitative food intake, and/or malabsorption. We believe that combined pharmacological and nutritional interventions, such as personalized therapy based on the specific needs of individual patients, can maintain cell metabolism hence aiding traditional standard therapy in CHF patients.

Data show that exogenous oral supplementation with a special mixture of individual essential AA tailored for human needs (EAAm) could be a valid therapeutic strategy to use in patients with CHF, in combination with conventional therapy to prevent protein malnutrition with protein wasting for the following reasons.

Firstly, EAAm could resolve malnutrition related to reduced nutrient gut absorption due to exocrine exhaustion of the pancreas caused by CHF. It is well known that the pancreas is the major consuming organ of AA. Furthermore, the pancreas plays a fundamental role in food digestion. However, digestion and food absorption need adequate synthesis and secretion of different enzymes.

This process uses enormous quantities of AA and energy at all meals. In patients with CHF, the exocrine efficiency of the pancreas is progressively reduced. As we have seen, this then causes a vicious circle, lower digestion of proteins, and lower availability of AA for digestive enzyme synthesis.

This condition leads to impaired digestion with a consequent reduction of plasma patterns of AA which are insufficient to promote protein synthesis. Interestingly, single AA are not digested. They are rapidly absorbed and immediately available in the blood for protein synthesis [16].

Secondly, EAAm are positive signals for maintaining muscle protein stores and so reducing IR. Indeed, AA inhibit changes to glucose transport as well as gluconeogenesis mediated by IR. In addition, at high physiological concentrations, AA activate various important phases of protein synthesis [17, 18]. Data show that AA are signals for the secretion of IGF-1 and -2 (insulin-like growth factor-1 and -2) [19]. IGF-1 is the somatomedin responsible for the main activation of growth hormone (GH), an anabolic hormone which promotes protein synthesis and counteracts IR.

Thirdly, EAAm modulate the effects of insulin on adipocytes enhancing glucose-induced desensitization of insulin-stimulated glucose transport and regulate the synthesis of FFA [20]. In particular this effect is mediated by glutamine.

Finally, it has recently been demonstrated that EAAm, including essential and branched ones as well as lysine, can maintain protein synthesis, support energy needs, and stimulate mitochondrial biogenesis [21]. These effects are due to the capability of AA to activate AMP-activated protein kinase mammalian target of rapamycin (mTOR) and nitric oxide synthase (NOS). These are important enzymes which stimulate cell life and development (including stem and endothelial cells), regulating energy production/use, protein synthesis, cell proliferation, antiapoptotic processes, and mitochondrial biogenesis [22, 23].

In addition to EAAm, other molecules have been investigated and are presently under investigation, as nutritional support for CHF patients in order to understand their role in avoiding protein disarrangement. A recent multicenter phase III clinical trial demonstrated that Amalirin (orally active ghrelin receptor antagonist) can reverse muscle wasting in cancer cachexia and has been proposed as a therapeutic agent for all stage of cachexia. The protein's anabolism can also be modulated by stimulation of selective androgen receptor modulators (SARMs) in humans and by activating type II receptor of Myostatin in both animal models and humans. Another animal study showed that the anabolic/catabolic transforming agent MT-102 reverses muscle wasting in rats [24].

## 3. Gut Flora Dysbiosis

The mammalian gastrointestinal tract contains about 100 trillion microorganisms called gut flora or microbiota. This is ten times greater than the total number of human cells in the body. For this reason, it is also called “the forgotten organ.” Microbiota benefits the host by exerting many crucial functions for host life. Indeed, microbiota regulates the guts' barrier function and motion, influences nutrient absorption, produces important metabolic intermediaries, including vitamins, and modulates local and systemic inflammation through innate immunity. Consequently, gut microbiota influences the metabolism of tissues outside the intestine. Thus, alterations of the composition and/or activity of microbiota, known as dysbiosis, as well as intestinal functions, influence the evolution of diseases independent of the original cause of the disease. Recently, gut microbiota dysbiosis has been proposed as an environmental factor capable of causing a catabolic state with consequent muscle wasting [25–27].

### 3.1. The Evidence

Recently, we demonstrated that CHF patients had significant qualitative and quantitative impairment of gut flora. Particularly, pathological species were present in the majority of CHF patients. Indeed, we found the massive presence of* Candida*,* Campylobacter*,* Shigella*,* Salmonella*, and* Yersinia* in the stools of CHF patients. The massive presence of this pathogenic contaminating flora had important pathophysiological effects on patients with CHF, because they significantly correlate with systemic inflammation and intestinal functions including permeability (authors' unpublished data).

### 3.2. The Consequence

Research shows that there are molecular reasons for the link between dysbiosis and systemic catabolism. Indeed, pathogens produce lipopolysaccharide (LPS) and other fungal or bacterial toxins which cause locally produced cytokines with consequent intestinal epithelial inflammation, which increases gut membrane permeability. This intestinal endothelial dysfunction allows the translocation of fungal or bacterial toxins from the gut to the circulating blood. In turn, this increases both circulation inflammatory molecules such as TNF-alfa and LPS. These stimulate inflammatory cells to produce catabolic molecules capable of causing muscular wasting, sarcopenia, and finally cachexia [28–30].

Recently, researchers have identified the enzymatic activities of protein effectors injected into host cells by pathogens such as* Shigella*,* Salmonella*, and* Yersinia*. This occurs through their type III secretion system, which modulates host innate immune responses. Specifically, bacterial acetyl-transferase modifies AA residues of cellular MAPK and I-KK-beta modulating inflammatory cell response and activating intracellular apoptosis [31]. The consequent state of chronic inflammation has been well documented in CHF patients and it is thought to be responsible for functional organs dysfunction and clinical deterioration [28].

The anatomical and functional alteration of the enterocytes induced by contaminating flora also influences the intestinal nutrient absorption and gastrointestinal mobility causing malnutrition. This aspect is particularly important in CHF patients because it is well known that malnutrition, with consequent muscular wasting and cachexia, is very common. Various hypotheses can be formulated to explain the massive presence of contaminating pathological microorganisms in the gut flora. The more accredited hypotheses consider local ischemia and indiscriminate antibiotic use as responsible of pathogens overgrowth. Local ischemia is due to CHF-mediated distribution of blood flow which underperfuses gut endothelium. Ischemia causes intramucosal acidosis, hypercapnia, and oxygen-free radical production. Moreover, CHF patients have neuroendocrine activation with norepinephrine increase. These conditions are all known to be activators of bacterial virulence and can also influence pathological bacteria overgrowth (authors' unpublished data). The overuse of antibiotics in the food chain may contribute to selecting antibiotic resistant pathogens, which then colonizes the intestine.

## 4. Therapies

Currently, there are no specific therapies to cure microbiota dysbiosis available. However, encouraging data suggest that gut dysbiosis can be modified by several means such as nutritional supplementation with specific nutrients (prebiotics) and/or live bacteria (probiotics) and/or local therapies with fecal microbiota transplant or colon hydrotherapy [32].

Fecal microbiota transplant (or stool transplant) is a process of transplanting fecal bacteria from healthy individuals into recipients. FMT involves restoring the colonic microflora by introducing healthy bacterial flora through the infusion of stools. Colon hydrotherapy is a technique which reduces waste material in the colon by using tubes in order to insert water, with or without specific therapy, into the colon via the rectum with special equipment [32].

## 5. Conclusive Remarks

CHF is a complicated multifactor disease with important social-economic impact. In spite of the recent development of new drugs and therapeutic strategies, CHF-related mortality and morbidity remain high. Recent evidence suggests that changes in organs such as skeletal muscle and gut flora play an important and independent role in CHF prognosis. Specific therapies need to be identified in the future in order to maintain the metabolic and functional homeostasis of these organs so that we can cure CHF patients better.
